# Whole-Genome DNA Methylation Analysis in *Brassica rapa* subsp. *perviridis* in Response to *Albugo candida* Infection

**DOI:** 10.3389/fpls.2022.849358

**Published:** 2022-06-23

**Authors:** Soodeh Tirnaz, Naomi Miyaji, Shohei Takuno, Philipp E. Bayer, Motoki Shimizu, Mst. Arjina Akter, David Edwards, Jacqueline Batley, Ryo Fujimoto

**Affiliations:** ^1^School of Biological Sciences, University of Western Australia, Crawley, WA, Australia; ^2^Graduate School of Agricultural Science, Kobe University, Kobe, Japan; ^3^Iwate Biotechnology Research Center, Kitakami, Japan; ^4^Department of Evolutionary Studies of Biosystems, SOKENDAI, The Graduate University for Advanced Studies, Hayama, Japan; ^5^Department of Plant Pathology, Bangladesh Agricultural University, Mymensingh, Bangladesh

**Keywords:** epigenetic, plant immunity, White rust, komatsuna, RNA sequencing, gene expression, whole genome bisulfite sequencing

## Abstract

DNA methylation is an epigenetic mark associated with several mechanisms in plants including immunity mechanisms. However, little is known about the regulatory role of DNA methylation in the resistance response of *Brassica* species against fungal diseases. White rust, caused by the fungus *Albugo candida*, is one of the most widespread and destructive diseases of all the cultivated *Brassica* species, particularly *Brassica rapa* L. and *Brassica juncea* (L.) Czern and Coss. Here, we investigate whole-genome DNA methylation modifications of *B. rapa* subsp. *perviridis* in response to white rust. As a result, 233 and 275 differentially methylated regions (DMRs) in the susceptible cultivar “Misugi” and the resistant cultivar “Nanane” were identified, respectively. In both cultivars, more than half of the DMRs were associated with genes (DMR-genes). Gene expression analysis showed that 13 of these genes were also differentially expressed between control and infected samples. Gene ontology enrichment analysis of DMR genes revealed their involvement in various biological processes including defense mechanisms. DMRs were unevenly distributed around genes in susceptible and resistant cultivars. In “Misugi,” DMRs tended to be located within genes, while in “Nanane,” DMRs tended to be located up and downstream of the genes. However, CG DMRs were predominantly located within genes in both cultivars. Transposable elements also showed association with all three sequence contexts of DMRs but predominantly with CHG and CHH DMRs in both cultivars. Our findings indicate the occurrence of DNA methylation modifications in *B. rapa* in response to white rust infection and suggest a potential regulatory role of DNA methylation modification in defense mechanisms which could be exploited to improve disease resistance.

## Introduction

*Brassica rapa* L., a diploid species (*n* = 10) within the Brassicaceae family, is comprised of a wide range of morphotypes including oil types such as *B. rapa* subsp. *oleifera*, rapiferous-type such as turnip (*B. rapa* subsp. *rapa*), and leafy types such as pak choi (*B. rapa* subsp. *chinensis*), Chinese cabbage (*B. rapa* subsp. *pekinensis*), and komatsuna (*B. rapa* subsp. *perviridis*) (Prakash and Hinata, 1980). These varieties are among the most widely commercially grown cultivars of *B. rapa* (Mar, 2012; Lv et al., 2020).

Diseases are one of the major threats to *B. rapa* production worldwide. White rust, caused by the biotrophic fungus *Albugo candida*, is one of the widespread and destructive diseases of several wild crucifers and all the cultivated *Brassica* species, particularly *Brassica juncea* L., Czern and Coss (AABB genome) and its progenitors *B. rapa* (AA genome). Yield losses have been reported as up to 60% in Polish or Turnip rape (*B. rapa* subsp. *oleifera*) in Canada (Petrie and Vanterpool, 1974), up to 89.8% in Indian mustard (*B. juncea*) in India (Lakra and Saharan, 1988), and up to 10% in Australia (Barbetti, 1981; Meena et al., 2014).

The initial symptom of white rust is the appearance of white- to cream-colored pustules on cotyledons, leaves, and/or the stem and is known as “local” infection, while the spread and development of disease in stems, pod and inflorescence, and formation of stagheads is known as systemic infection and results in significant yield losses (Verma and Petrie, 1980). On delicate vegetable types of *B. rapa*, such as pak choi, Chinese cabbage, and komatsuna, even a slight white rust infection can cause all the production unmarketable (Santos et al., 2006). Biological races of *A. candida* have been classified based on host specificity and race 7 is known to affect *B. rapa*. However, there is not an absolute classification between the races, as race 7 also affects *Brassica napus* L. and *B. juncea* (Tanhuanpaa and Vilkki, 1999; Adhikari et al., 2003). These race composition of the *A. candida* pathogen makes identification of resistance sources against *A. candida* challenging. Different management strategies have been employed for white rust management including crop rotation, weed removal, and fungicide application. However, the best approach is the cultivation of resistant cultivars (Asif et al., 2017). The need for resistant cultivars is even more crucial for vegetable types of crops, such as *B. rapa*, as fungicide application carries a risk of fungicide residue remaining in food (Santos et al., 2006).

The complexity of identifying resistance sources against white rust is even more intensified by the complication of underlying regulatory mechanisms of resistance responses. One of the main regulatory mechanisms of resistance response is through epigenetic modifications, including DNA de/methylation, chromatin remodeling, and histone modification. DNA methylation, one of the key epigenetic marks with a proven regulatory role in plant immunity, refers to the addition of a methyl group to the cytosine bases of DNA to form 5-methylcytosine (Colot and Rossignol, 1999). In plants, methylation of cytosine bases is observed in the context of symmetric CG and CHG and asymmetric CHH (where H = A, C, or T) (Henderson and Jacobsen, 2007). DNA methylation is one of the key factors for genome stability and transcriptome regulation (Fujimoto et al., 2012). DNA methylation status is highly affected by several factors including environmental conditions (e.g., biotic and abiotic stresses), tissue type, and growth stage (Choi and Sano, 2007; Fujimoto et al., 2008a, 2012; Kawakatsu and Ecker, 2019). Several studies are revealing the pattern of DNA methylation changes associated with resistance/tolerance improvement under both biotic and abiotic stresses in various plant species; for example, tolerance improvement under salinity stress in rice (Karan et al., 2012), *Medicago truncatula* (Yaish et al., 2018) and wheat (Zhong et al., 2009), under drought stress in rice (Wang et al., 2010, 2016), and under heat stress in *B. rapa* (Liu et al., 2018) and resistance improvement in watermelon against *cucumber green mottle mosaic virus* (CGMMV) (Sun et al., 2019), in rice against *Magnaporthe grisea* (Li et al., 2011; Deng et al., 2017), and in tomato against *Tomato yellow leaf curl Sardinia virus* (TYLCSV) (Mason et al., 2008).

DNA methylation through transcriptome reprogramming regulates the plant response to environmental stresses (Elhamamsy, 2016; Tirnaz and Batley, 2019a,b). Stress-induced DNA methylation changes can occur in any context (i.e., CG, CHG, and CHH) and genomic regions [e.g., promoters, gene bodies, and transposable elements (TEs)] (Dowen et al., 2012; Wang et al., 2014a), which add complexity to understand its exact role in gene regulation and defense mechanisms. For example, gene body methylation in the CG context has been shown to have a positive correlation with gene expression in common bean, soybean, and rice (Li et al., 2012; Kim et al., 2015; Wang et al., 2017a). In addition, in rice, hypo- and hyper-methylation in the promoters of nucleotide-binding site leucine-rich repeat (NLRs) occurs under pathogen attack, that is, *M. grisea* and *Xanthomonas oryzae* pv. *oryzae*, resulted in transcriptome reprogramming and a resistance response (Akimoto et al., 2007; Li et al., 2011; Deng et al., 2017).

The importance of DNA methylation modifications in plant resistance and its potential in improving breeding programs has been emphasized (Springer and Schmitz, 2017; Tirnaz and Batley, 2019a,b), highlighting the importance of taking DNA methylation modifications into account when breeding toward resistance improvement. Therefore, we investigated genome-wide DNA methylation modifications in susceptible and resistant *B. rapa* subsp. *perviridis* komatsuna cultivars in response to *A. candida* to better understand the regulatory role of DNA methylation in resistance response.

## Materials and Methods

### Plant Materials

**Figure 1:**
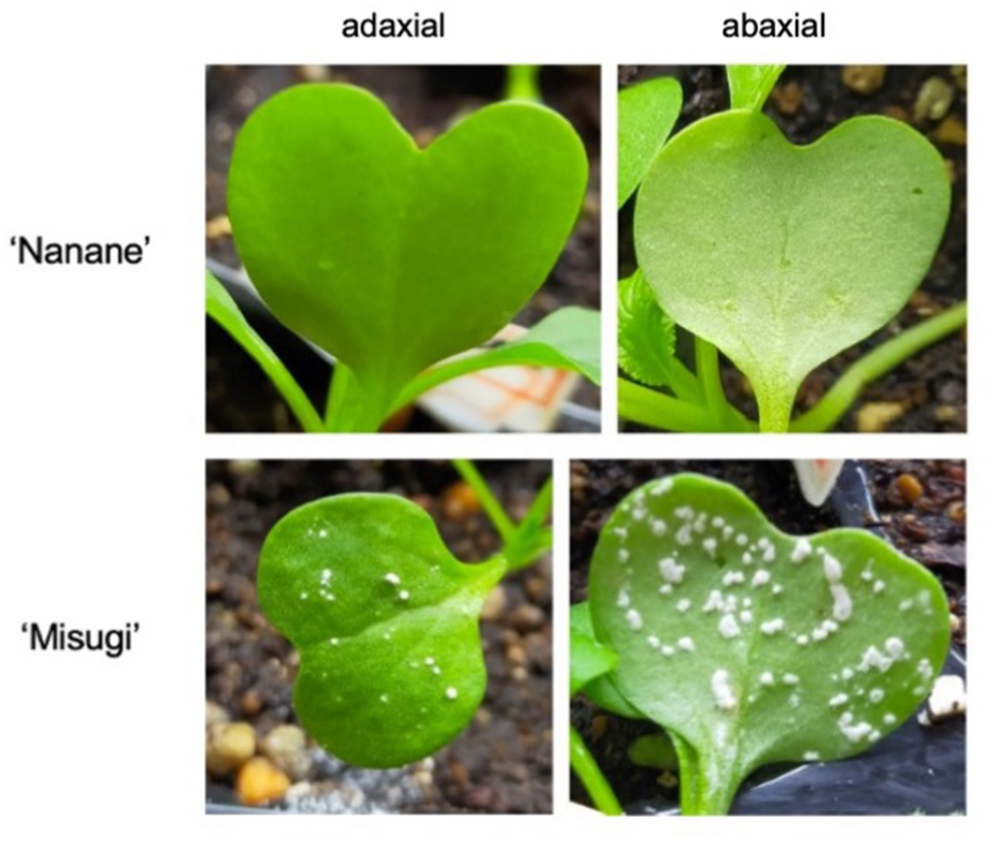
Phenotype caused by *A. candida* 10 days after inoculation at the seedling stage of komatsuna (*Brassica rapa* subsp. *perviridis*). Susceptible cultivar: “Misugi,” Resistant cultivar: “Nanane.”

Two *B. rapa* subsp. *perviridis* cultivars (komatsuna) were selected, one susceptible “Misugi” (Sakata Seed Corporation, Japan) and one resistant “Nanane” (Takii & Co., Ltd., Japan) to *A. candida* Mibuna isolate WMB01 (Figure 1). Seeds of “Misugi” and “Nanane” cultivars were sown on soil and kept under 16 h light and 8 h dark at 21°C. Seven-day-old plants were inoculated by spraying the *A. candida* (WMB01) with a concentration of 1 × 10^5^ zoosporangium/ml. Mock inoculation with water was also performed for control samples. To ensure successful inoculation, plants were incubated in a dark growth chamber for 24 h at 22°C with 100% humidity. Then, the plants were moved to a growth chamber and kept under growth conditions of 16 h light and 8 h dark at 21°C, with regular irrigation. For DNA methylation and gene expression studies, one cotyledon of each plant was harvested after 72 h of inoculation, snap-frozen in liquid nitrogen, and stored at −80°C until further use.

### DNA Extraction and Bisulfite Sequencing Library Construction

Genomic DNA of infected and control samples of susceptible and resistant cultivars was extracted using the CTAB method (Murray and Thompson, 1980). The construction of the bisulfite sequencing library was performed by the BGI Genomics company. In brief, genomic DNA (1,000 ng) was sheared to 200–300 bp using sonication (Covaris^®^ LE220). DNA end-repair, 3′-dA overhang, ligation of methylated sequence adaptors, and bisulfite treatment were performed using the ZYMO EZ DNA Methylation-Gold kit (ZYMO RESEARCH, USA) according to the manufacturer's instructions. The qualified library was sequenced paired-end (150 bp) on an Illumina HiSeqXTEN System. Two biological replicates were used in this study.

### Whole-Genome Bisulfite Sequencing Analysis

The reads from whole-genome bisulfite sequencing (WGBS) were trimmed using Trimmomatic-0.39 and quality control was performed using FastQC. Trimmed and high-quality reads were mapped to the reference genome of *B. rapa* V3 (Zhang et al., 2018) using Bowtie2 version 2.2.5 and Bismark-v0-14.3 (Krueger and Andrews, 2011). PCR duplicates were removed as one of the reads, which align to the same position, and are randomly retained by using duplicate_bismark script in the bismark package. To calculate the methylation level of CG, CHG, and CHH contexts, the number of methylated and unmethylated reads were extracted at each cytosine position using a bismark methylation extractor script with a paired-end parameter. The methylation level at each cytosine was calculated by dividing the number of methylated cytosine reads by the total number of reads. A binomial test (Lister et al., 2008) was performed for the classification of methylated and unmethylated cytosine sites. The sequence context-specific error rates of bisulfite conversion were estimated from the mapping results of the unmethylated phage genome and used for the binomial test. The test was applied only to the cytosine sites with greater than or equal to 3 WGBS coverage. A significant cut-off of two-tailed *P* < 0.01 was used to detect methylated cytosine.

### Differentially Methylated Regions Analysis

To detect the differentially methylated regions (DMRs), the genome was divided into 500 bp windows with no overlap. The total number of cytosines in each context (CG, CHG, and CHH) in a given window were calculated and based on the distribution frequency, and windows containing ≥ 10 cytosines in all CG, CHG, and CHH contexts were kept for downstream analysis. The methylation level of CG, CHG, and CHH was calculated in each window by dividing the number of methylated cytosines in each given context by the total number of cytosines in the given context. To identify the DMRs in each cultivar between control and infected samples, an *F*_*ST*_-like approach was performed. *F*_*ST*_ statistics are widely used in population genetics to measure the level of population differentiation (Holsinger and Weir, 2009). Here, first, highly differentiated windows between the biological replicates were removed and the remaining windows were used for *F*_*ST*_ calculation, where the methylation level of a certain window was denoted as *X1*: methylation level of a given cultivar under control condition for replicate 1, *X2*: methylation level of the given cultivar under control condition for replicate 2, *X3*: methylation level of the given cultivar after infection with *A. candida* replicate 1 and *X4*: methylation level of the given cultivar after infection with *A. candida* for replicate 2; the variance between replicates (*V*) and absolute methylation difference between treatments (δ) was then calculated as


$$\begin{array}{l} V=\max\left(\left|X1-X2|,|X3-X4\right|\right)\\ \delta =|\frac{X1+X2}{2}-\frac{X3+X4}{2}| \end{array}$$

where the max (*X, Y*) function is to take *X*, if *X* > *Y*, otherwise to take *Y*. Using *V* and δ value, the relative methylation difference in the given window was calculated as


$$\begin{array}{l} F_{ST}=1-\frac{V}{\delta } \end{array}$$

*F*_*ST*_ was calculated for each methylation context (i.e., CG, CHG, and CHH) separately in both resistant and susceptible cultivars. To detect the regions with maximum methylation difference between control and infected samples while ensuring the minimum differences between biological replicates only, windows with top 1% *F*_*ST*_ and top 1% δ values were assigned as putative DMRs and used for downstream analysis. DMRs were associated with their proximal genes where DMRs were located at the gene body (i.e., between the start and stop codon including all exons and introns of a gene), and/or located up to 2 kb upstream and downstream of genes. Then, DMR-associated genes were used for gene ontology (GO) enrichment analysis. GO enrichment analysis was performed using the R package topGO (v 2.40.0). All the heatmaps for comparative analysis of DNA methylation levels were constructed using the R package ComplexHeatmap (V 2.2.0).

To verify the WGBS results, we picked four regions, including hyper- and hypo-methylated DMRs in both cultivars. A total of 500 ng of DNA was fragmented by sonication and the fragments were ~300–800 bp in length. MethylCode Bisulfite Conversion Kit (Thermo Fisher Scientific, Inc, USA) was used for chemical bisulfite reaction and PCR was performed using bisulfite-treated DNAs as templates. PCR conditions were 95°C for 2 min followed by 40 cycles of 95°C for 30 s, 55°C for 30 s, and 72°C for 30 s. Amplified PCR fragments were gel-purified using GENECLEAN III Kit (MP Biomedicals, USA) and cloned into pGEM-T Easy vector (Promega Co., USA). Ten independent clones were sequenced. Primers used for bisulfite sequencing are listed in Supplementary Table 1.

### RNA Extraction and RNA Sequencing Analysis

Total RNAs of infected (72 h after *A. candida* inoculation) and control samples of resistant and susceptible cultivars were extracted using SV Total RNA Isolation System (Promega). RNA sequencing was performed using paired-end Nextseq500 (75 bp read length). The number of clean reads and the percentage of mapped reads are shown in Supplementary Table 2. Low-quality reads were filtered using FASTX-Toolkit v. 1.4.5 and HISAT2 was used to align the filtered reads to the reference genome of *B. rapa* V3 (Zhang et al., 2018). The expression levels (fragments per kilo-base per million—FPKM) were scored using Cuffdiff. Differentially expressed genes were also identified based on two criteria of two-fold difference (|log 2 ratio| ≥ 1.0) and 95% confidence. Differentially expressed genes located close to DMRs were also detected and their protein sequences were searched against Pfam, SMART, and PRINTS databases using InterProScan (https://www.ebi.ac.uk/interpro/) for domain identification.

Seven genes were used for the validation of RNA-seq results. cDNA was synthesized from 500 ng total RNA using the ReverTra Ace^®^ qPCR RT Master Mix with gDNA Remover (TOYOBO Co., Ltd., Japan). The specificity of the primer set of each gene was first tested by electrophoresis of RT-PCR amplified products using QuickTaq^®^HS DyeMix (TOYOBO) on 1.5% agarose gel in which single products were observed. RT-PCR conditions were 94°C for 2 min followed by 35 cycles of 94°C for 30 s, 55°C for 30 s, and 68°C for 30 s. The absence of genomic DNA contamination was confirmed by the PCR of no RT control. Real-time RT-PCR (qPCR) was performed using a LightCycler 96 (Roche Molecular Systems, Inc., USA). cDNA was amplified using FastStart Essential DNA Green Master (Roche). qPCR conditions were 95°C for 10 min followed by 40 cycles of 95°C for 10 s, 60°C for 10 s, and 72°C for 10 s, and a melting program of 65–97°C at 0.1°C/s. After amplification cycles, each reaction was subjected to melt temperature analysis to confirm the presence of single amplified products. The relative expression level of each gene relative to *ACTIN* (*Bractin*) was automatically calculated using automatic CQ calling according to the manufacturer's instructions (Roche) (Fujimoto et al., 2006a). The data presented are the average and standard error of three biological and experimental replicates. The primer sets are listed in Supplementary Table 1.

## Results

### Analysis of Whole-Genome Bisulfite Sequencing

**Figure 2:**
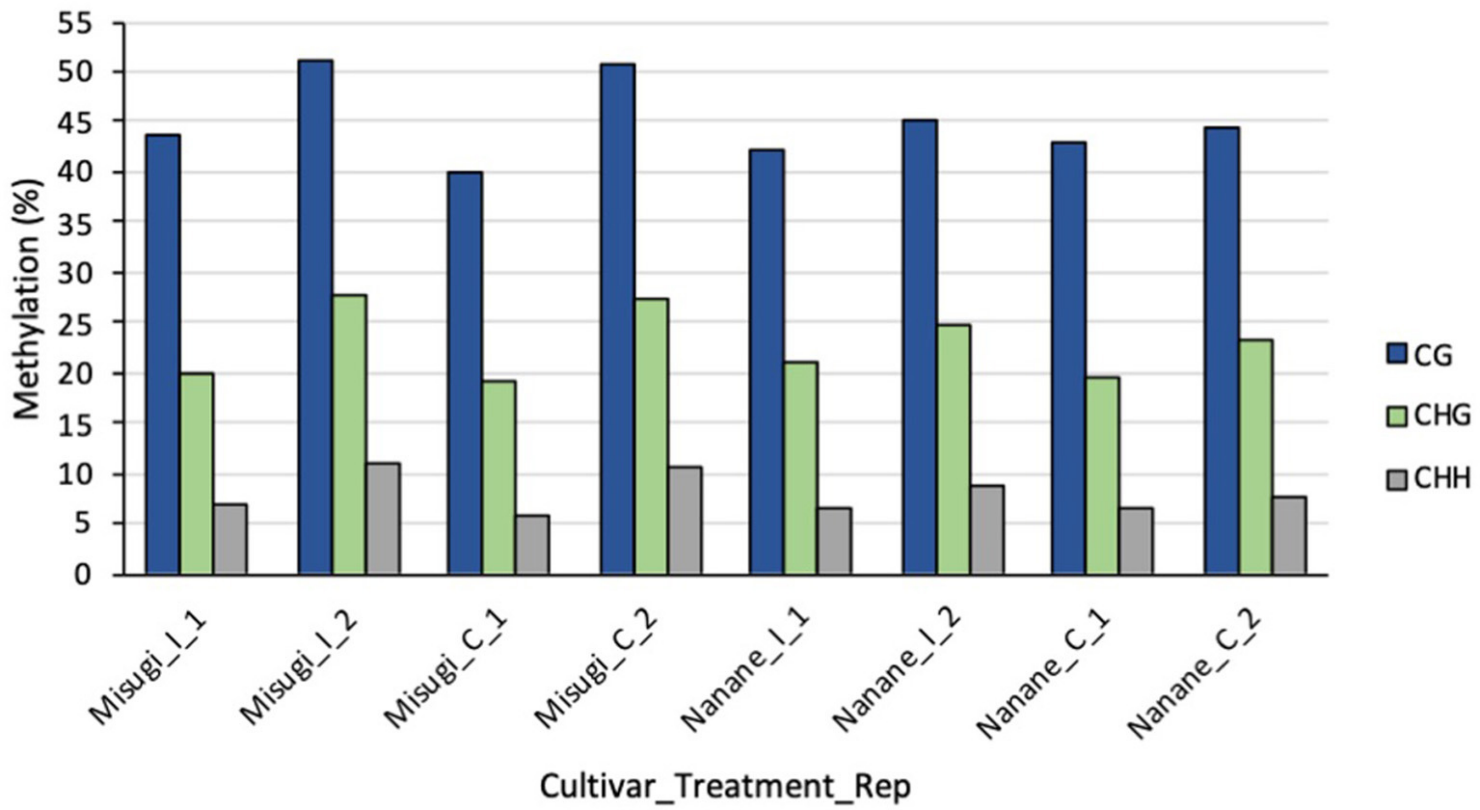
Percentage of methylated cytosine in each context (CG, CHG, and CHH) in *B. rapa* cultivars “Misugi” (susceptible) and “Nanane” (resistant) for control (_C) samples and infected (_I) samples with *A. candida* for replicate one (_1) and two (_2).

WGBS was performed to detect the single-based resolution and relative amount of 5-methylcytosines (5-mCs) changes across the genome of susceptible (“Misugi”) and resistant (“Nanane”) cultivars of *B. rapa* in response to *A. candida*. The high-quality reads were mapped to the *B. rapa* reference genome (Zhang et al., 2018) (Table 1). The mapping efficiency of all samples was between 35.9 and 43.7% (Table 1). The bisulfite conversion error of CG, CHG, and CHH of all samples was between 0.002135 and 0.004833 (Table 1). In both “Misugi” and “Nanane,” DNA methylation occurs predominantly at the CG sites, followed by CHG and CHH, ranging between 40 and 51%, 19 and 27%, and 5 and 10% of sites being methylated, respectively (Figure 2).

**Table 1:** Mapping results and bisulfite conversion error of *B. rapa* cultivars “Misugi” (susceptible) and “Nanane” (resistant).

**Sample**	**Total read pairs**	**Paired-end alignments**	**Mapping efficiency**	**Error rates of bisulfite conversion**		
	**(RP)**	**with a unique best hit**		**CG**	**CHG**	**CHH**
Misugi_I_1	41217472	17466392	42.40%	0.002135	0.002227	0.002333
Misugi_I_2	45530660	16662331	36.60%	0.003629	0.003695	0.004184
Misugi_C_1	41161501	18200401	42.20%	0.002249	0.002352	0.002496
Misugi_C_2	42164051	15150569	35.90%	0.004208	0.004318	0.004833
Nanane_I_1	41128620	17500829	42.60%	0.002265	0.002394	0.002553
Nanane_I_2	49694053	20128844	40.50%	0.003297	0.003400	0.003687
Nanane_C_1	41060329	17930058	43.70%	0.002367	0.002492	0.002617
Nanane_C_2	47673367	19292368	40.50%	0.003621	0.003723	0.003945

*Control (_C) samples and infected (_I) samples with A. candida for replicate one (_1) and two (_2)*.

### Identification of Differentially Methylated Regions

**Figure 3:**
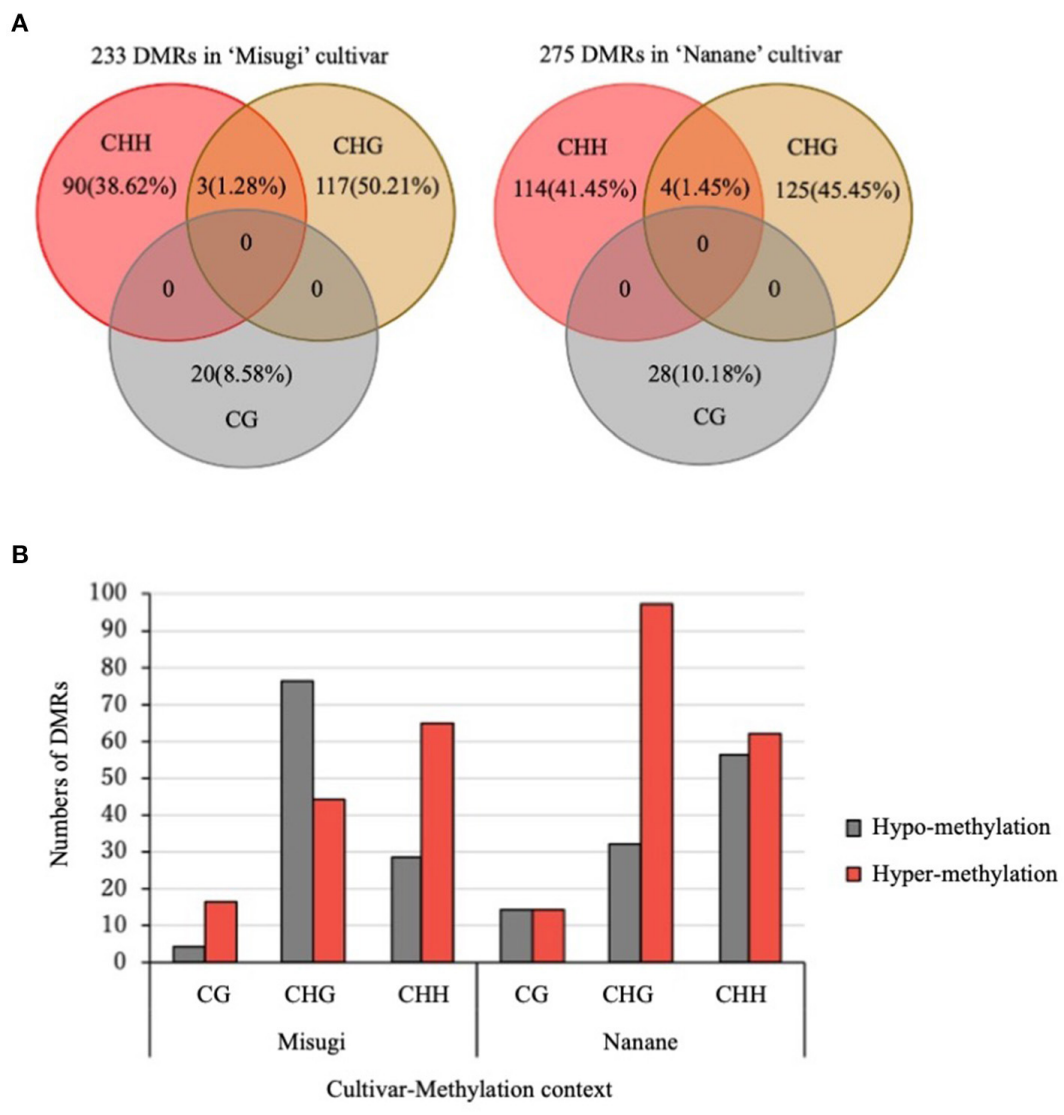
Differentially methylated regions (DMRs) between control cotyledons and cotyledons infected with *A. candida* of *B. rapa* cultivars “Misugi” (susceptible) and “Nanane” (resistant). **(A)** Total number and percentage of putative CG, CHG, and CHH DMRs. **(B)** Methylation status of 233 and 275 DMRs in “Misugi” (susceptible) and “Nanane” (resistant), respectively.

DMRs of each sequence context (CG, CHG, and CHH) were detected between control and infected samples of “Misugi” and “Nanane.” Regions were assigned as putative DMRs where maximum methylation difference occurs between control and infected samples while having the minimum differences between biological replicates. In total, 233 and 275 DMRs were detected in the susceptible cultivar “Misugi” and the resistant cultivar “Nanane,” respectively (Figure 3A and Supplementary Table 3). No overlapping DMRs were found in all three cytosine contexts between “Misugi” and “Nanane.” We defined the heterochromatic region as having more than 0.4 of the density of TEs. Approximately 30% of DMRs were found in heterochromatic regions in both “Misugi” and “Nanane,” and more than 40% of CHG DMRs were in heterochromatic regions (Supplementary Table 3).

In both cultivars, despite the higher percentage of methylated cytosine at CG sites through the genome, the number of CG DMRs (“Misugi:” 20, “Nanane:” 28) was noticeably lower than DMRs for CHG (“Misugi:” 120, “Nanane:” 129) and CHH (“Misugi:” 93, “Nanane:” 118) (Figure 3A and Supplementary Table 3).

The comparative analysis of the methylation level and status of DMRs (i.e., hypo- and hyper-methylation) showed in both cultivars, more than half of DMRs were hyper-methylated, 53.64 and 62.90% in “Misugi” and “Nanane,” respectively. In the susceptible cultivar “Misugi,” most of the CG (80.00%) and CHH (69.89%) DMRs were hyper-methylated, while the majority of CHG DMRs (63.33%) were hypo-methylated. In the resistant cultivar “Nanane,” half of the CG DMRs (50.00%) and CHH (52.54%) were hypo- and hyper-methylated and CHG (75.19%) was predominantly hyper-methylated after infection with the pathogen (Figure 3B, Supplementary Figure 1, and Supplementary Table 3). Bisulfite sequencing confirmed the reliability of the performed WGBS (Supplementary Figure 2).

### Detection of Differentially Methylated Regions Associated With Genes

**Figure 4:**
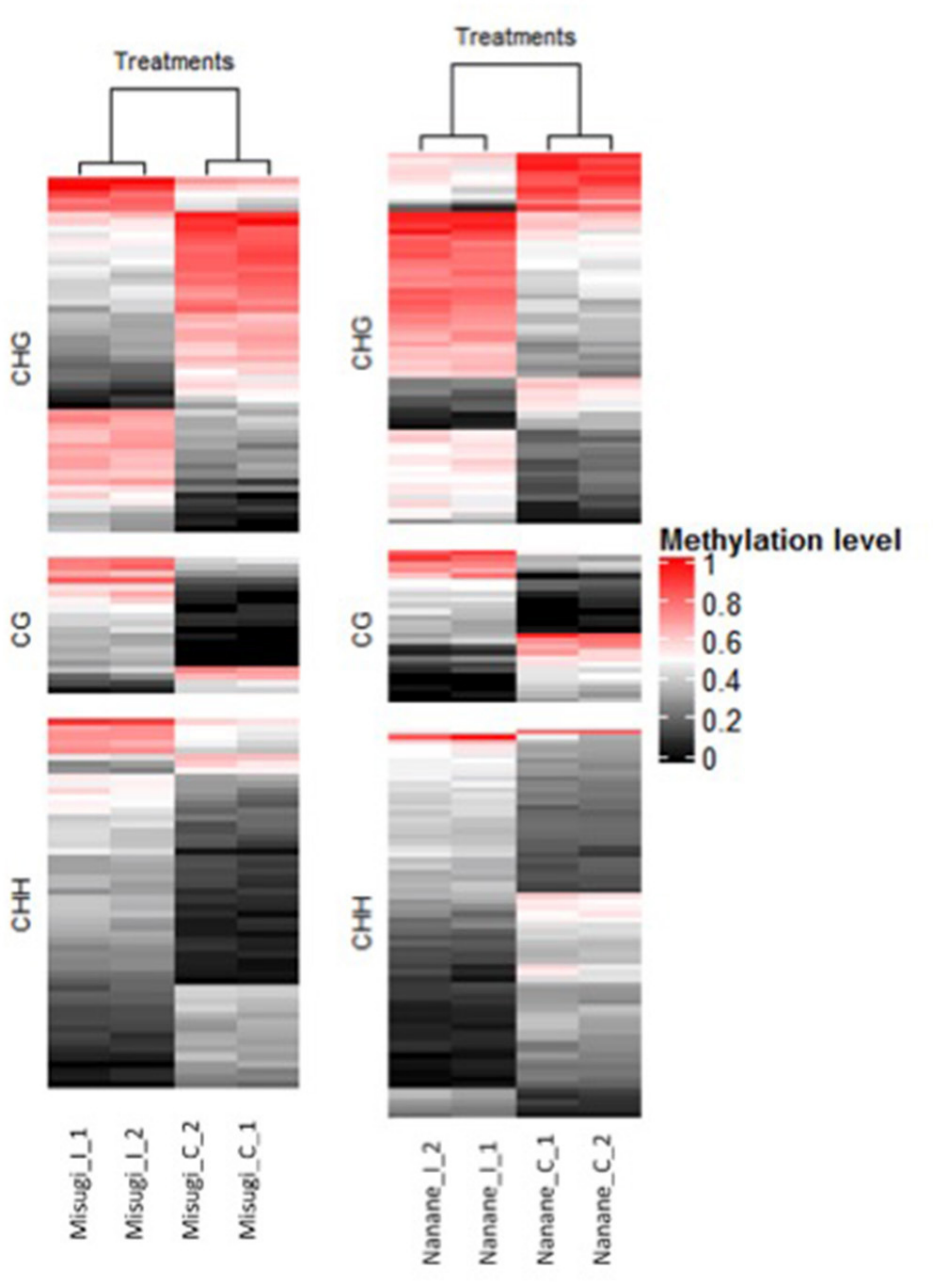
Heatmap of methylation levels of 155 and 126 differentially methylated regions (DRMs) - associated genes, respectively, in *B. rapa* cultivars “Misugi” (susceptible) and “Nanane” (resistant) for control (_C) samples and infected (_I) samples with *A. candida* for replicate one (_1) and two (_2). Each line represents one DMR. Methylation level 1 means all the cytosines of a given context are methylated in the region.

Genes associated with DMRs were screened where DMRs were located in gene bodies (i.e., from the start codon to stop codon) and/or up to 2 kb up/downstream of genes. Out of the 233 DMRs in the susceptible cultivar “Misugi,” 126 (54.07%) were proximate to 136 genes (CG: 21, CHG: 56, CHH: 59) and in the resistant cultivar “Nanane,” out of 275 DMRs, 155 (56.36%) were proximate to 178 genes (CG: 29, CHG: 72, CHH: 77) (Figure 4, Table 2, and Supplementary Table 3). In both cultivars, the majority of genes were linked to the CHH DMRs (Figure 4).

**Table 2:** Differentially methylated regions (DMRs) associated with gene body, upstream, and downstream of genes in *B. rapa* cultivars “Misugi” (susceptible) and “Nanane” (resistant).

		**Upstream-2 kb**			**gene body**			**Downstream-2 kb**			**Total**
		**Hypo**	**Hyper**	**Total**	**Hypo**	**Hyper**	**Total**	**Hypo**	**Hyper**	**Total**	
“Misugi”	CG	1	2	3	3	14	17	0	1	1	21
				14.28%			80.95%			4.76%	
	CHG	9	8	17	12	8	20	10	9	19	56
				30.35%			35.71%			33.92%	
	CHH	4	12	16	12	12	24	3	16	19	59
				27.11%			40.67%			32.20%	
Total		14	22	36	27	34	61	13	26	39	136
		10.29%	16.17%	26.47%	19.58%	25.00%	44.85%	9.55%	19.11%	28.67%	
“Nanane”	CG	5	3	8	7	10	17	3	1	4	29
				27.58%			58.62%			13.79%	
	CHG	8	17	25	2	16	18	12	17	29	72
				34.72%			25.00%			40.27%	
	CHH	21	11	32	5	11	16	13	16	29	77
				34.72%			20.77%			37.66%	
Total		34	31	65	14	37	51	28	34	62	178
		19.10%	17.41%	36.51%	7.86%	20.78%	28.65%	15.73%	19.10%	34.83%	

**Figure 5:**
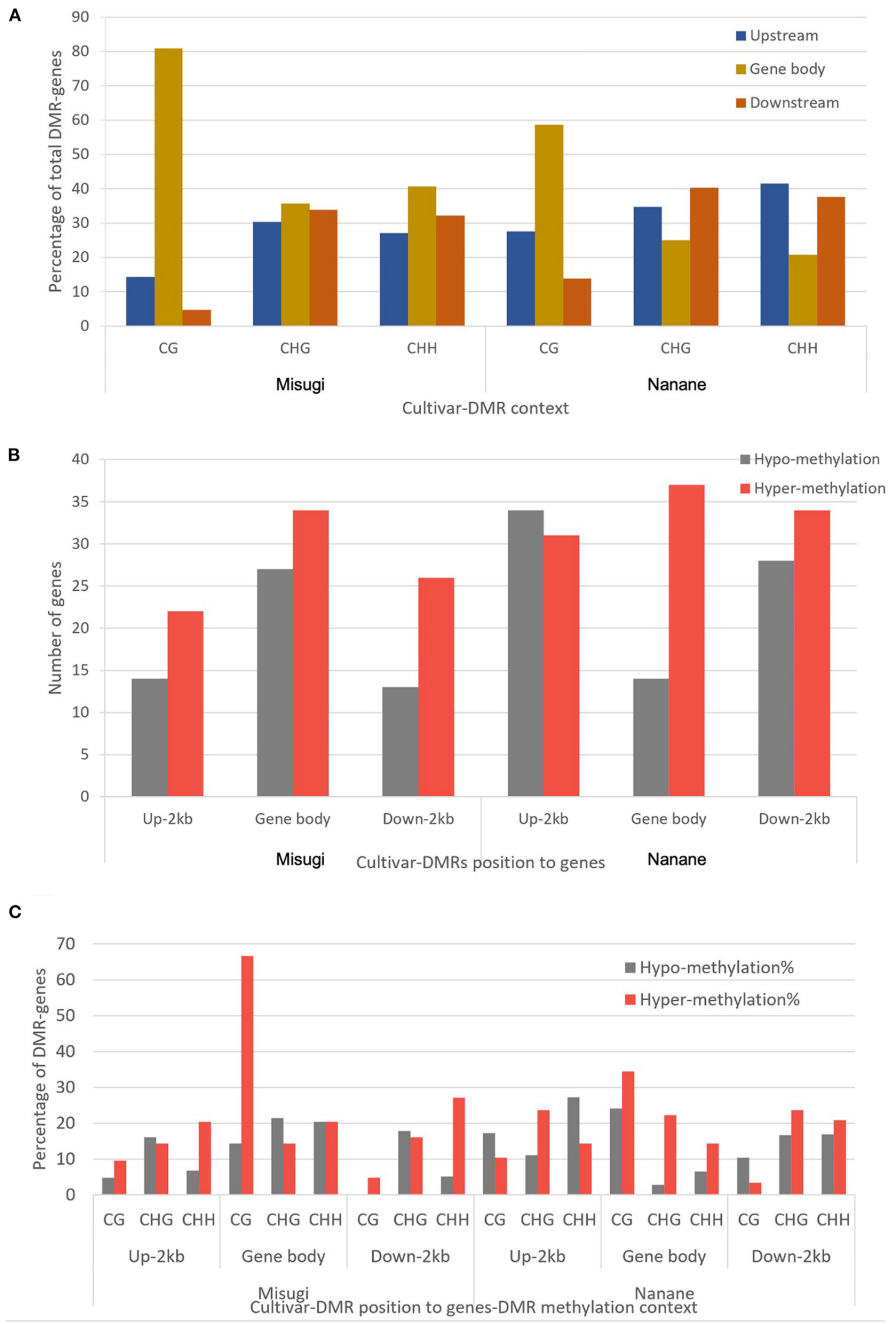
Proximate location and methylation status of differentially methylated regions (DMRs) in *B. rapa* cultivars “Misugi” (susceptible) and “Nanane” (resistant). **(A)** Percentage of 136 and 178 genes associated with DMRs in “Misugi” (susceptible) and “Nanane” (resistant), respectively. **(B)** Total number of genes associated with hypo and hyper DMRs at each proximate location (i.e., gene body and/or up to 2 kb upstream and downstream of genes). **(C)** Percentage of DMR-associated genes with hypo- and hyper-methylated CG, CHG, and CHH DMRs.

We then identified DMR locations relative to genes. In “Misugi” (susceptible cultivar), 61 out of 136 DMRs (44.85%) were located in gene bodies, which also included the highest number of all three DMRs' contexts (CG: 17, CHG: 20, CHH: 24). In “Nanane” (resistant cultivar), 65 out of 178 (36.51%) DMRs were located upstream of genes with the highest number of CHH (32) and CHG (25) DMRs and 62 out of 178 (34.83%) DMRs were located downstream of genes with the highest number of CHG [29 (40.27%)] and CHH [29 (37.66%)] DMRs (Figure 5A and Table 2). Despite the difference in CHG and CHH DMRs distribution between the two cultivars, in both cultivars, most of the CG DMRs are located in gene bodies (“Misugi:” 80.95%, “Nanane:” 58.62%) (Figure 5A and Table 2).

In both “Misugi” and “Nanane,” 59.52 and 58.06% of DMRs associated with genes were hyper-methylated, respectively. In both cultivars, the hyper-methylation was predominant in DMRs within the gene bodies and downstream of genes; however, in the upstream of genes, the majority of DMRs were hyper-methylated in “Misugi” (16.17%) and hypo-methylated (19.10%) in “Nanane” (Figure 5B and Table 2).

In “Misugi,” CG DMR-genes tended to be hyper-methylated, dominantly located within the gene body (66.66%). No hypo-methylated CG DMRs were identified in the downstream of the genes (Figure 5C and Table 2). CHH DMRs tended to be hyper-methylated at the upstream and downstream of the genes (Figure 5C and Table 2). In “Nanane,” CG DMRs tended to be hypo-methylated at the upstream and downstream of the genes, while CG DMRs tended to be hyper-methylated within the gene body (Figure 5C and Table 2). In addition, CHG and CHH DMRs were dominantly hyper-methylated in the gene body (Figure 5C and Table 2). In the upstream region, CHG DMRs tended to be hyper-methylated, while CHH DMRs tended to be hypo-methylated (Figure 5C and Table 2).

### Functional Analysis of Genes Associated With DMRs

**Figure 6:**
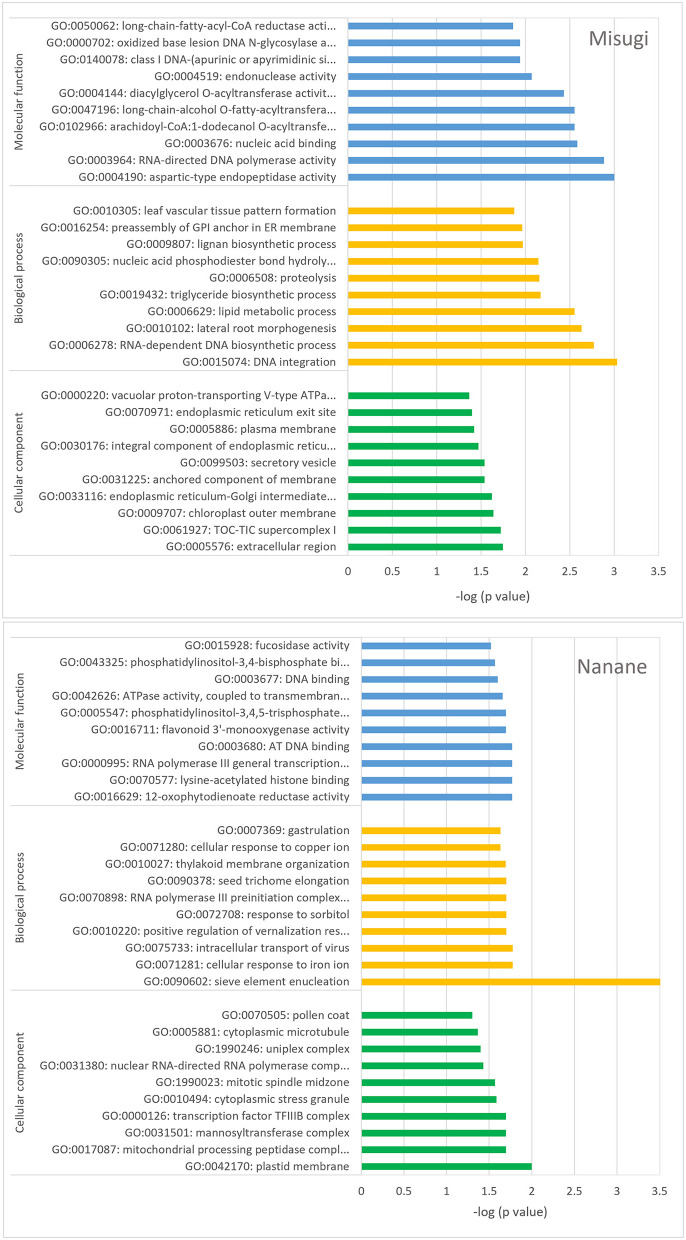
Gene ontology (GO) enrichment analysis. Genes associated with differentially methylated regions (DMRs) between control cotyledons and cotyledons infected with *A. candida*, in *B. rapa* cultivars “Misugi” (susceptible, 136 genes) and “Nanane” (resistant, 178 genes) were used. The top 10 classes of three categories (biological process, molecular function, and cellular component) are exhibited.

GO enrichment analysis of genes associated with DMRs indicates the involvement of genes in all three GO categories, that is, biological process, molecular function, and cellular component (Figure 6 and Supplementary Table 4). DMR-associated genes in “Misugi” were highly enriched for biological process and and molecular function. The top two highly enriched (*P* < 0.05) classes for the biological process were DNA integration (GO: 0015074) and RNA-dependent DNA biosynthetic process (GO: 0006278) and top two of highly enriched (*P* < 0.05) terms for molecular function were aspartic-type endopeptidase activity (GO: 0004190) and RNA-directed DNA polymerase activity (GO: 0003964) (Figure 6 and Supplementary Table 4). In “Nanane,” DMRs-associated genes were highly enriched for the plastid membrane (GO: 0042170) from the cellular component category and for the sieve element enucleation (GO: 0090602) from the biological process category (Figure 6 and Supplementary Table 4). DMR genes were also compared against *B. rapa* resistance gene analogous (RGAs), which were reported by Tirnaz et al. (2020a), and the results showed that DMR genes in “Misugi” and “Nanane” included two receptor-like kinases (RLKs) genes (Supplementary Table 3).

### Differentially Methylated Regions Associated With Transposable Elements

**Figure 7:**
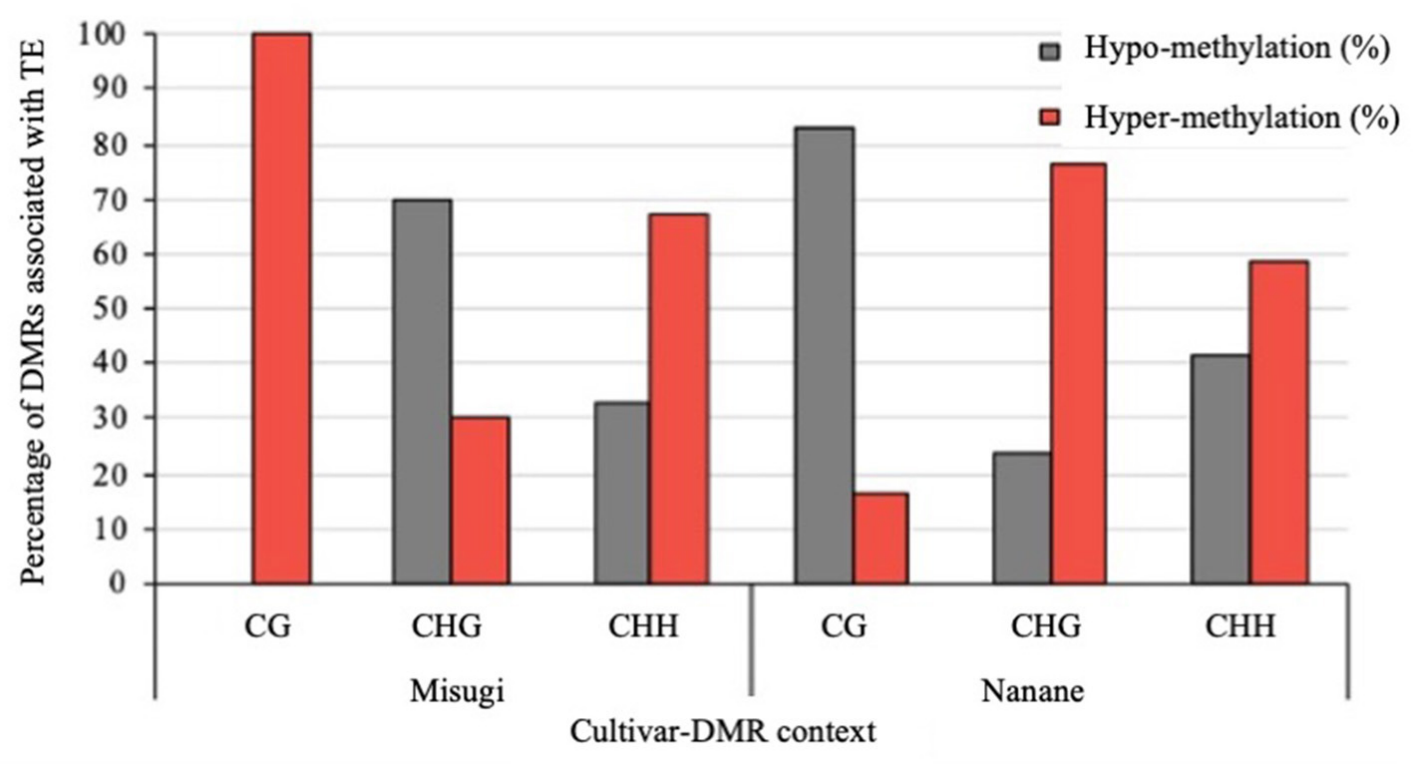
Percentage of differentially methylated regions (DMRs), between control cotyledons and cotyledons infected with *A. candida*, associated with transposable elements (TEs) at each methylated context in *B. rapa* cultivars “Misugi” (susceptible) and “Nanane” (resistant).

DNA methylation is one of the main regulatory mechanisms controlling TEs (Frost et al., 2005; Fujimoto et al., 2008b; Sasaki et al., 2011), movements, and activity throughout the genome (Tirnaz and Batley, 2019a). In this regard, here we investigated the occurrence of DMRs within TEs. In the susceptible cultivar (“Misugi”), 117 out of 233 DMRs (50.21%) were associated with TEs (DMR-TEs), and in “Nanane,” 129 out of 275 DMRs (46.90%) were associated with TEs (Supplementary Table 3). About 40% of DMRs associated with TEs were located in the heterochromatic regions in “Misugi” and “Nanane” (Supplementary Table 3). Consistent with other studies (Cokus et al., 2008; Li et al., 2012; Regulski et al., 2013; Song et al., 2013; Wang et al., 2015), we found TE methylation in all three contexts (Supplementary Figure 3). From total DMRs in each context in “Misugi,” Ten percentage of CG DMRs, 52.50% of CHG DMRs, and 55.91% of CHH DMRs were associated with TEs. Similarly, in “Nanane,” a low percentage of CG DMRs (21.42%) were associated with TEs. However, as opposed to “Misugi,” CHG DMRs (55.81%) showed a higher percentage of TE association than CHH DMRs (43.22%). In addition, the results revealed that in “Misugi,” 100% of CG-DMR associated with TEs were hyper-methylated, while the majority of CG-DMR-associated TEs in “Nanane” (83.33%) were hypo-methylated (Figure 7). DMRs in CHG context also showed the opposite trend of methylation between the two cultivars, where in “Misugi,” the large number of CHG-DMR associated with TEs were hypo-methylated (69.84%) and in “Nanane,” they were hyper-methylated (76.38%) (Figure 7). In both cultivars, the higher percentage of CHH-DMR associated with TEs were hyper-methylated (Figure 7). We also found 17 DMRs (CG: 1, CHG: 8, CHH: 8) and 30 DMRs (CG: 3, CHG: 13, CHH: 14) in association with TEs located at up to 2 kb upstream of genes in “Misugi” and “Nanane,” respectively (Supplementary Table 3). In addition, 20 DMRs (CG: 1, CHG: 7, CHH: 12) and 16 DMRs (CG: 2, CHG: 7, CHH: 7) in association with TEs located within the genes in “Misugi” and “Nanane,” respectively (Supplementary Table 3). About 35 and 18% of DMR-TEs were *Gypsy*-type in “Misugi” and “Nanane,” respectively, and half of CHH-DMR associated with TEs were *Gypsy*-type in “Misugi” (Supplementary Table 5).

### Association Between DNA Methylation and Gene Expression

Here, RNA-seq analysis on control and infected cotyledons (72 h after *A. candida* inoculation) of “Misugi” and “Nanane” cultivars were performed. The results showed that, in total, 4,104 and 2,157 genes were differentially expressed in “Misugi” (down: 1,979, up: 2,125) and “Nanane” (down: 1,112, up: 1,045), respectively (Supplementary Table 6). In total, seven genes were selected and the expression levels of these genes in control and infected samples of “Misugi” and “Nanane” cultivars were examined by qPCR. There was a high correlation (*r* = 0.97 in “Misugi,” *r* = 0.96 in “Nanane”) of the ratio of expression levels between the control and infected samples of these seven genes observed between qPCR analysis and RNA-seq data (Supplementary Table 7). The association study of differentially expressed genes with DMRs showed that, in total, 13 genes were associated with DMRs in “Misugi” (8 genes) and “Nanane” (5 genes). The methylation context of DMRs between the two cultivars appeared to be opposite, while CG and CHH contexts were predominated in “Misugi” and CHG context was predominated in “Nanane” (Table 3). Four (1 in “Nanane” and 3 in “Misugi”) differentially expressed genes overlapped with DMR-TEs (Supplementary Table 3).

**Table 3:** The status of 13 differentially expressed genes (up/downregulation) and their association with differentially methylated regions (DMRs) in *B. rapa* cultivars “Misugi” (susceptible) and “Nanane” (resistant).

**Cultivars**	**Gene**	**Expression status**	**DMR methylation status**	**DMR-Type**	**DMR location to gene**
“Misugi”	BraA03g060690.3C	Down	Hyper	CHH	Upstream
	BraA06g000420.3C	Up	Hypo	CG	Gene body
	BraA06g019710.3C	Down	Hyper	CG	Gene body
	BraA08g005490.3C	Down	Hyper	CHH	Downstream
	BraA08g025160.3C	Up	Hyper	CHH	Downstream
	BraA09g021410.3C	Up	Hyper	CHH	Upstream
	BraA09g060940.3C	Up	Hyper	CG	Upstream
	BraA10g002450.3C	Down	Hyper	CHH	Gene body
“Nanane”	BraA01g007120.3C	Up	Hypo	CHG	Downstream
	BraA03g007980.3C	Down	Hyper	CHG	Downstream
	BraA07g015170.3C	Up	Hypo	CHH	Upstream
	BraA08g012250.3C	Down	Hyper	CHG	Upstream
	BraA09g010440.3C	Down	Hyper	CHG	Gene body

In total, 22 domains were identified across 10 genes and no domains have been identified for 3 genes (Supplementary Table 8). Some of the domains are reported to be involved in defense mechanisms such as flavin-dependent monooxygenases (FMOs) (Mishina and Zeier, 2006; Thodberg and Jakobsen Neilson, 2020) and type III polyketide synthase-like protein (PKS) (Tanjung et al., 2020). Similarly, genes containing haloacid dehalogenase-like hydrolase and Proline-rich domains were also expressed in sweet orange in response to citrus blight (Fu et al., 2019). In addition, the *Arabidopsis thaliana* orthologs of these 13 genes have been identified, and 8 genes out of 13 had orthologs in *A. thaliana* (Supplementary Table 8). *A. thaliana* orthologs, including AT1G55690 (Sec14p-like phosphatidylinositol transfer family protein), AT1G03870 (FASCICLIN-LIKE ARABINOOGALACTAN 9, FLA9), and AT1G24510 (CCT5, CHAPERONIN CONTAINING T-COMPLEX POLYPEPTIDE-1 SUBUNIT 5), have multiple roles in plant growth, development, and signaling pathways (Kamal et al., 2020; Wu et al., 2020), but their involvement in response to pathogens is not well-understood.

## Discussion

DNA methylation is involved in defense mechanisms, but little is known about its regulatory role in *B. rapa* against fungal disease. WGBS was performed on white rust susceptible (“Misugi”) and resistant (“Nanane”) cultivars of komatsuna (*B. rapa* subsp. *perviridis*) after inoculation with *A. candida* and inoculation with water (mock inoculation). The highest methylation level was detected at CG sites followed by CHG and CHH sites after mock and *A. candida* inoculation. Similarly, in wheat, after mock and pathogen [*Blumeria graminis* f. sp. *tritici* (*Bgt*)] inoculation, DNA methylation occurs in descending order of CG (~87%), CHG (~57%), and CHH (~1.6%) sites (Geng et al., 2019). Independent of pathogen attack, genome-wide analysis of several plants is also evidenced that DNA methylation occurs in the same pattern, such as in *B. rapa* subsp. *pekinensis* (CG: 36.5%, CHG: 13.4%, CHH: 5.3%) (Takahashi et al., 2018a) and *B. rapa* subsp. *oleifera* (CG: 52.4%, CHG: 31.8%, CHH: 8.3%) (Chen et al., 2015).

In *B. napus* in response to blackleg disease in a resistant cultivar (“Sturt”), more promoters of resistance genes (23.66%) were differentially methylated in comparison to the susceptible cultivar “Westar” (14.42%) (Tirnaz et al., 2020b). In this study, similar numbers of DMRs were observed in the susceptible cultivar (“Misugi”) and the resistant cultivar (“Nanane”), and no overlapped DMRs were found between the two cultivars, indicating that DMRs by *A. candida* infection are cultivar specific. Most of the methylated CG sites were heavily methylated, while methylated CHG and CHH sites had low methylation levels in pre-infected plants of *B. rapa* (Takahashi et al., 2018a). The enzyme responsible for each cytosine context is different; CG and CHG methylation is mainly mediated by maintenance DNA methylase, while CHH methylation is mainly mediated by *de novo* DNA methylation by RNA-directed DNA methylation (Fujimoto et al., 2012). A large number of DMRs were detected in CHG and CHH contexts in both cultivars. The higher number of CHG and CHH DMRs indicates that CHG and CHH sites are the most affected loci during *A. candida* infection, which may be due to the difference in robustness of DNA methylation between CG and non-CG methylation. Similarly, methylation at CHH sites has been reported to be the mainly affected in wheat against powdery mildew disease (Geng et al., 2019), in *Citrullus lanatus* against cucumber green mottle mosaic virus (CGMMV) (Sun et al., 2019), and in *B. napus* against blackleg disease (Tirnaz et al., 2020b). There were differences in the ratios between the number of DMRs of hyper- and hypo-methylation by *A. candida* infection between cultivars. CG and CHH methylation tended to be hyper-methylated in “Misugi,” but this was not observed in “Nanane.” CHG methylation tended to be hypo-methylated in “Misugi,” while hyper-methylated in “Nanane.” Further study is required to determine whether differences in DMRs between cultivars are associated with differences in disease resistance.

In both cultivars, one-third of CHG and CHH DMRs were located in the region upstream of genes. In general, the methylation level of upstream (promoter) regions results in gene regulation where hypo-methylation results in upregulation and hyper-methylation results in downregulation, and these have been previously reported among defense-related genes. For example, in rice hypo-methylation of the promoter region of *Xa21G*, a *X. oryzae* pv. *oryzae* resistance gene, resulted in a high level of gene expression and subsequently the resistant phenotype to the pathogen (Akimoto et al., 2007). Similar to biotic stress, promoter hypo-methylation due to abiotic stress, such as salinity and drought, induces upregulation of abiotic stress response genes (Choi and Sano, 2007; Wang et al., 2014b; Yaish et al., 2018). In the genus Arabidopsis, natural variation of the expression levels of *FWA* genes is also negatively associated with the DNA methylation level, especially with the CHH methylation level in the region just upstream of the transcription start site (Fujimoto et al., 2008a, 2011). Whereas, promoter hypo-methylation does not necessarily increase gene expression and results in resistance response. For instance, in rice partial demethylation in the promoter region of the resistance gene (*Pib*) compromises the resistance response to *M. grisea* due to its downregulation (Li et al., 2011). In addition, we found most of the CG DRMs were located in the gene bodies in both cultivars, and in “Misugi,” CG DRMs were hyper-methylated. Several studies have shown that gene body methylation predominantly occurs in the CG context and it has been shown that there is a positive correlation with gene expression, for example, in *A. thaliana* (Cokus et al., 2008), Chinese cabbage (*B. rapa*) (Takahashi et al., 2018a,b), cassava (*Manihot esculenta*) (Wang et al., 2015), soybean (*Glycine max*) (Song et al., 2013), maize (*Zea mays*) (Regulski et al., 2013), and rice (*O. sativa*) (Li et al., 2012). However, gene body methylation in CHG and CHH contexts shows a negative correlation with gene expression in tomato (González et al., 2011), *A. thaliana* (You et al., 2012), Chinese cabbage (Takahashi et al., 2018a), and common bean (Richard et al., 2018). These evidences that the sequence context of methylation within the gene body are important for the regulation of gene expression. GO enrichment analysis showed that some enriched GO classes were reported to be involved with defense mechanisms. For example, in *A. thaliana*, the overexpression of an aspartic-type endopeptidase activity (GO: 0004190) encoding gene causes resistance to *Pseudomonas syringae* pathogen (Xia et al., 2004). In tomato, in response to leaf miner (*Tuta absoluta*), differentially expressed genes between control and infested samples were also enriched for plastid membrane (GO: 0042170) (Manzo, 2016). In *A. thaliana*, sieve elements (GO: 0090602) show involvement in transferring long-distance nutrients (sugar) and signals in the phloem (Furuta et al., 2014). Sieve elements such as sugar transporters showed involvement in triggering signaling pathways in the host plant upon pathogen attack (Rolland et al., 2006; Doidy et al., 2012; Walerowski et al., 2018), and the over-represented GO class of sieve elements among DMR-associated genes in the resistant cultivar “Nanane” might be involved in the resistance response against *A. candida* infection. However, in this study, hyper- or hypo-methylation by *A. candida* infection in either the promoter regions or gene bodies was not associated with the change of gene expression in both lines. We need to examine whether the resistant line-specific changes of DNA methylation modification could be one of the strategies for resistant response.

In both cultivars, we evidenced modification of TE methylation at all three sequence contexts and almost half of the DMRs located within TEs. About 40% of DMR-TEs were located in heterochromatic regions and four of the DMR-TEs overlapped with differentially expressed DMR genes. TEs are dynamic elements of genomes and are involved in various mechanisms of gene regulation and evolution including intron generation, exon generation, change of local genome structure, alternative splicing, and transcriptome reprogramming (Fujimoto et al., 2006b; Barbazuk et al., 2008; Hirsch and Springer, 2017; Akter et al., 2021). However, the extent to which this mechanism is affected under plant-pathogen interaction is unclear. Biotic stress-induced hypo-methylation can increase TEs mobility within disease-related genes and affect their expression level (Biémont and Vieira, 2006), for example, in maize upon pathogen (*Fusarium graminearum*) challenge CACTA-like transposable element (TE1) inserted into a resistance gene (*qRfg1*) and suppress gene pathogen-induced expression, resulting in disease susceptibility (Wang et al., 2017b). DNA methylation modification of TEs at upstream of the genes also proved to play a regulatory role under pathogen pressure in *A. thaliana* against *Fusarium oxysporum* (Le et al., 2014) and in rice against *M. grisea* (Deng et al., 2017). Our results confirmed the dynamic of DNA methylation modification within TEs in *B. rapa* as a result of *A. candida* infection. The expression level of four genes neighboring DMR-TEs was changed but the association was not significant enough to support the hypothesis of the occurrence of gene expression modification up on methylation changes in nearby TEs.

We found that only 13 (8 “Misugi” and 5 “Nanane”) DMR-associated genes were differentially expressed. In *A. thaliana*, change of DNA methylation by pathogen infection is partially responsible for transcriptional control (Dowen et al., 2012). Hyper- or hypo-methylation in mutants of genes involved in DNA methylation did not always lead to a change of their transcription (Zhang et al., 2006; Zilberman et al., 2007). Differentially expressed genes varied with the time of infection (Miyaji et al., 2017). In the case of nematode inoculation in rice roots, hypo-methylation of CHH sites in the promoter regions at 3-day post-inoculation (dpi) was not associated with upregulation of genes, however, they showed association with genes upregulated at 7 dpi (Atighi et al., 2020). Although the results showed both DNA methylation modifications and gene expression regulation accrued during pathogen attack, the low number of differentially expressed genes associated with DMRs can be due to the small effect of DNA methylation change on gene expression or temporal differences in the effect of DNA methylation changes on gene expression. We need to examine additional time courses of *A. candida* infection to understand gene regulation through DNA methylation modification. It is also suggested to examine the inheritance of DMRs, and whether DMRs can generate a new epi-allele.

This study by investigating the dynamics of DNA methylation of susceptible and resistant cultivars of *B. rapa* subsp. *perviridis* against white rust disease enhance our knowledge of DNA methylation modification in response to pathogens, which can lead the direction of future studies to better understand the role of DNA methylation in plant immunity.

## Data Availability Statement

The original contributions presented in the study are publicly available. This data can be found here: DDBJ, PRJDB12782, and PRJDB12789.

## Author Contributions

STi, NM, STa, and RF conceived this study. NM and MA performed laboratory procedures. STi, NM, STa, PB, and MS carried out data analysis. STi and NM wrote the manuscript. DE, JB, STa, and RF supervised data analysis and provided critical revisions to the manuscript. All authors read and approved the manuscript.

## Funding

This work was funded by the Grant-in-Aid for JSPS Research Fellow to NM (18J20027) and grants from the Project of the Bio-oriented Technology Research Advancement Institution (Research program on development of innovative technology) to RF (30029C).

## Conflict of Interest

The authors declare that the research was conducted in the absence of any commercial or financial relationships that could be construed as a potential conflict of interest.

## Publisher's Note

All claims expressed in this article are solely those of the authors and do not necessarily represent those of their affiliated organizations, or those of the publisher, the editors and the reviewers. Any product that may be evaluated in this article, or claim that may be made by its manufacturer, is not guaranteed or endorsed by the publisher.
