# Supplementary material for: Whole-Genome DNA Methylation Analysis in Brassica rapa subsp. perviridis in Response to Albugo candida Infection
Source: Front Plant Sci. 2022 Jun 23;13:849358. doi: 10.3389/fpls.2022.849358 (PMC9261781; doi:10.3389/fpls.2022.849358)
Supplement: Supplementary file 1 [file Data_Sheet_1.docx]

Supplementary Material

## Supplementary Figures


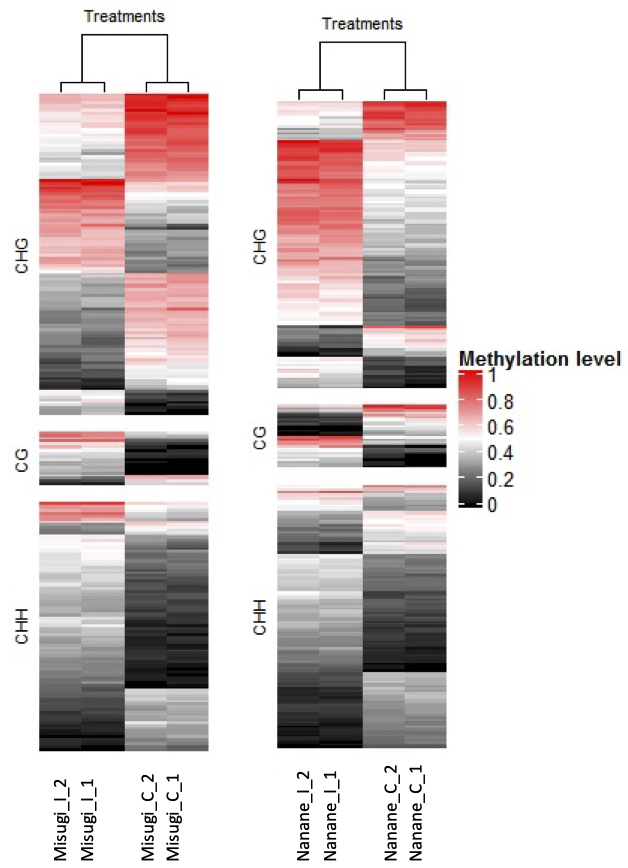


**Supplementary Figure 1.** Heatmap of methylation levels of 275 and 233 differentially methylated regions (DMRs), respectively in *B. rapa* cultivars ‘Nanane’ (resistant) and ‘Misugi’ (susceptible) for control (_C) samples and infected (_I) samples with A. candida for replicate one (_1) and two (_2). Each line represents one differentially methylated region (DMR). Methylation level 1 means all the cytosines of a given context are methylated in the region.

**Supplementary Figure 2.** Validation of DNA methylation states by bisulfite sequencing. Ten clones from each bisulfite-treated template were analyzed. The CHH hyper- and hypo-methylated regions in ‘Misugi’ and ‘Nanane’ detected by WGBS analysis were selected. Numbers in parentheses indicate the number of cytosine sites. Samples for bisulfite sequencing is independent from samples used for WBGS.


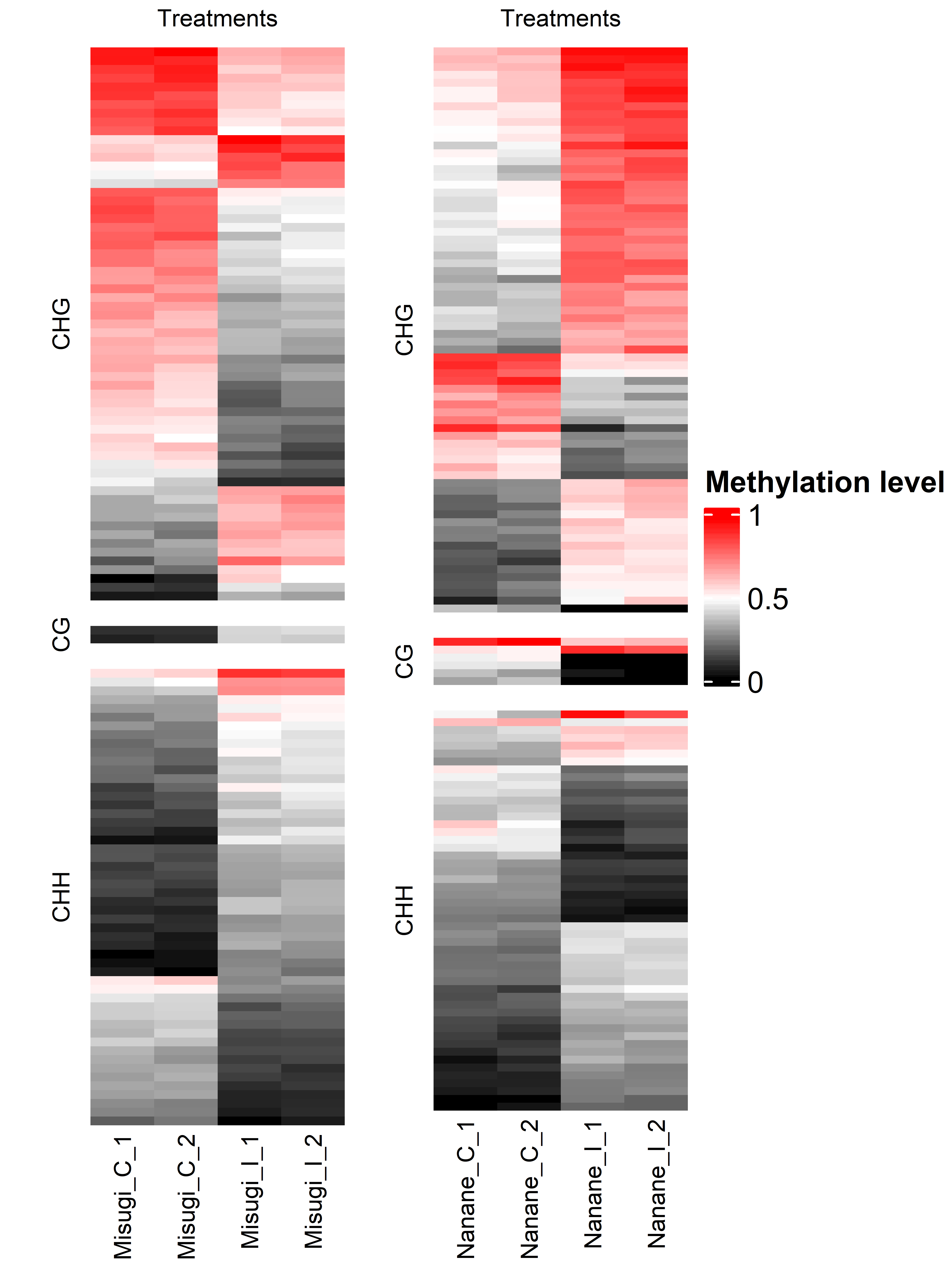


**Supplementary Figure 3.** Heatmap of methylation levels of differentially methylated regions (DMRs) associated with transposable elements (TEs) in *B. rapa* cultivars ‘Nanane’ (resistant) and ‘Misugi’ (susceptible) for control (_C) samples and infected (_I) samples with *A. candida* for replicate one (_1) and two (_2). Each line represents one differentially methylated region (DMR). Methylation level 1 means all the cytosines of a given context are methylated in the region.
